# Semiautomatic three-dimensional ultrasound renal volume segmentation in pediatric hydronephrosis: interrater agreement and correlation to conventional hydronephrosis grading

**DOI:** 10.1007/s00247-025-06249-8

**Published:** 2025-05-06

**Authors:** Michael Esser, Ilias Tsiflikas, James R. Jago, Laurence Rouet, Alexander Stebner, Jürgen F. Schäfer

**Affiliations:** 1 Department of Diagnostic and Interventional Radiology, Universitätsklinikum Tübingen, Hoppe-Seyler-Str.3, 72076 Tübingen, Germany; 2 Philips Healthcare, Ultrasound General Imaging, Bothell, WA, United States; 3 Philips Health Technology Innovation, Paris, France

**Keywords:** Hydronephrosis, Imaging, three-dimensional, Kidney diseases, Pediatrics, Ultrasonography

## Abstract

**Background:**

Two-dimensional (D) assessment of renal volume underestimates the actual value and shows high interobserver variability. Limited data exist on innovative 3-D ultrasound (US) technique for the evaluation of pediatric hydronephrosis.

**Objective:**

To assess the interrater agreement of kidney volume segmentation by 3-D US with a matrix array transducer in children with hydronephrosis and to compare the 3-D metrics to conventional hydronephrosis grading.

**Materials and methods:**

We prospectively acquired 48 renal volumes in 45 patients with hydronephrosis by freehand 3-D US (6–1 MHz volumetric sector array, electronic rotation; median age, 4 years; 1 month to 16 years). Semi-automated kidney segmentation was performed by two independent readers providing volumes for total kidney (renal capsule), dilated collective system, renal parenchyma (renal capsule-collective system) and hydronephrosis index (renal parenchyma/capsule). Interrater agreement was evaluated with Bland–Altman plots, intraclass correlation coefficient (ICC) and Dice similarity coefficients. The maximum calibre of renal pelvis was measured and hydronephroses were morphologically classified grade 1–4.

**Results:**

Interrater agreement for renal capsule, collective system, hydronephrosis index, and renal parenchyma was good to excellent with ICC of 0.94, 0.87, 0.83 and 0.92 respectively (*P* < 0.001 each). Median Dice was 0.90 (capsule), 0.77 (collective system) and 0.88 (parenchyma). There was a positive correlation between conventional hydronephrosis grading and ultrasonic hydronephrosis index and between renal pelvis diameter and collective system volume (*P* < 0.001 both).

**Conclusion:**

Semiautomatic 3-D US volumetric analysis has a high degree of interrater agreement and enables reliable assessment of renal parenchymal volume in hydronephrosis. Volumes of the collective system and hydronephrosis index correlate with the extent of hydronephrosis.

**Trial registration:**

Trial registration number, DRKS00022772; date of registration, 07/31/2020.

**Graphical Abstract:**

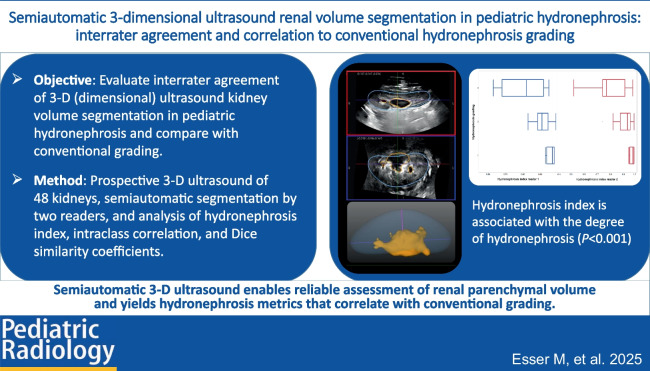

## Introduction

In children with hydronephrosis, renal parenchymal volume is a key indicator for prognosis and disease monitoring. It serves as a surrogate marker for renal function and parenchymal damage, suggesting the need for invasive diagnostic and therapeutic intervention [1]. Sonographic volume assessment, a non-invasive and radiation-free method, reflects actual organ size and nephron mass (including glomeruli and tubules) [2]. While conventional sonographic evaluation of the renal volume has been well-established for decades, it primarily relies on 2-dimensional (2-D) measurements using the simplified ellipsoid formula. This approach is known to underestimate the true volume and is associated with a high interobserver variability [3–5]. Although this method is easy to apply in normal, non-hydronephrotic kidneys, accurate assessment becomes more challenging in the presence of hydronephrosis, associated malformation, or post-surgical deformities, especially considering the dynamic nature of renal growth and the variability of pelvic dilation [1, 3].

Measurements such as anteroposterior diameter, which uses simple threshold values, and calyceal dilation are limited because they are influenced by the hydration status and urinary bladder filling. These measurements often fail to reflect the actual degree of hydronephrosis, particularly in cases with variable pelvic anatomy [6]. Existing radiology grading systems are based on subjective criteria and are therefore not recommended for therapeutic decision-making. As 2-D ultrasound (US) measurements may be inaccurate in anatomically complex kidneys, there is currently no imaging gold standard for determining neither the severity of hydronephrosis nor the parenchymal volume [1, 7].

Today, 3-D US has become an established tool in several specialties. Since the early 2000 s, this technique has increasingly been applied in the pediatric population showing improved accuracy for renal volume assessment with a low interobserver variability [3, 7–9]. However, there is limited data on its use for estimating pediatric hydronephrosis, where it might offer added value compared to cases with preserved anatomy. Previous studies of renal 3-D US in adults did not include hydronephrosis [10–13] and/or were limited to preselected clinical subgroups [8].

With recent technological advances, matrix array transducers now enable near-instant freehand acquisition of volumetric data without requiring an electric motor or position sensor. These transducers have been shown to considerably reduce volumetric errors caused by motion artifacts (which may occur in children who cannot hold their breath or only for a short time). Initially used in adults, including a cadaver study and comparisons of normal organ volumes with CT and renal function data [11, 12], matrix array transducers were later applied in children for renal volume measurements in polycystic kidney disease [8]. To date, their use in pediatric hydronephrosis has only been reported in a feasibility study involving eight kidneys for a new segmentation model [14]. To the best of our knowledge, no further clinical data exist regarding the use of a matrix array transducer in children with hydronephrosis.

Our study aimed to evaluate the interrater agreement of renal volume segmentation using 3-D US with a matrix array transducer in children with hydronephrosis, and to compare the resulting metrics to conventional hydronephrosis grading.

## Materials and methods

This prospective single center study was approved by the local ethics committee. Written informed consent was obtained from all legal, as well as from participating children aged 11 years and older. All study procedures were conducted in accordance with the Guidelines for Good Clinical Practice and ethical standards as laid down in the 1964 Declaration of Helsinki and its later amendments or comparable ethical standards. The authors had complete control of the data.

### Study population

Between March and September 2021, all patients under 17 years of age who were referred to our center for pediatric urology consultation with a clinical indication for US of the urinary tract and present hydronephrosis were included in the study unless they or their legal guardians declined to participate.

### Three-dimensional ultrasound acquisition and evaluation

Initial routine 2-D US was performed by a pediatric radiologist with 7 years of experience (M.E.) using an EPIQ 5G US device (Philips Medical Systems, Bothell, WA). The maximum anteroposterior diameter of the renal pelvis was measured on transverse 2-D B-mode images. Hydronephrosis was classified into grade 1–4 following the original Onen- 2007 system to ensure comparability with previous studies [1].

**Fig. 1:**
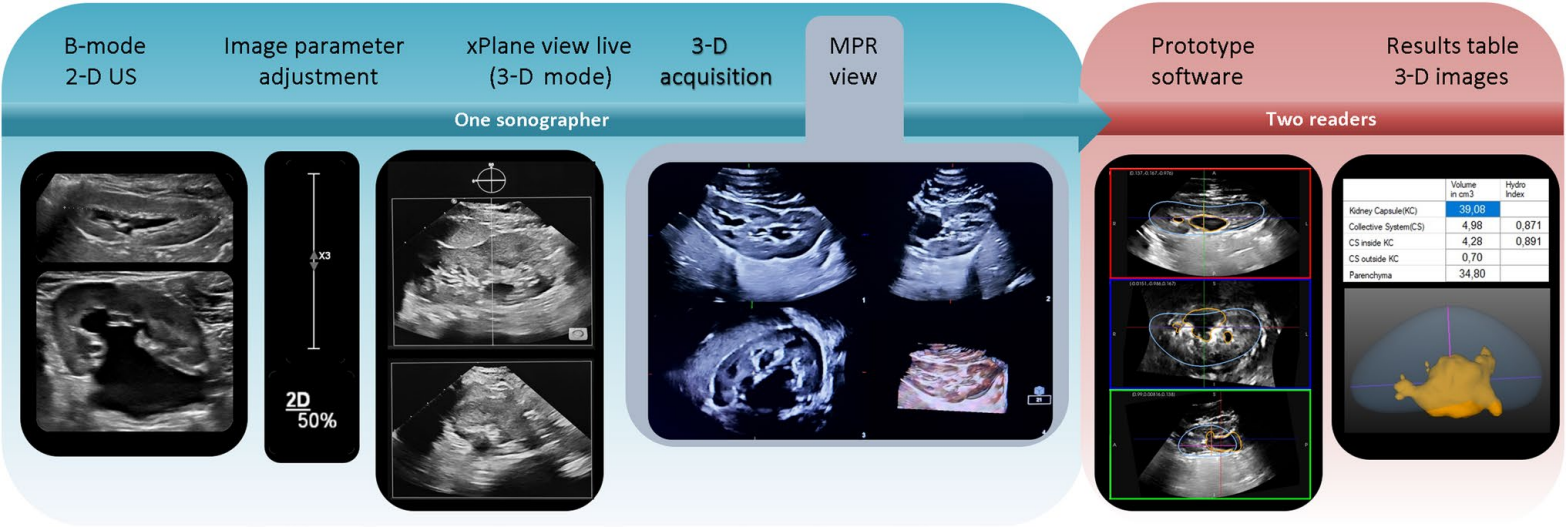
Workflow of the study. Image parameters were adjusted based on the conventional 2-dimensional (D) B-mode ultrasound. Afterward, the 3-D mode display was used to show live side-by-side-image including a sagittal and axial view of the kidney at the same time. After 3-D volume acquisition the multiplanar reformatted (MPR) view highlights the structure in three orthogonal planes and a volume-rendered image. The segmentation was performed independently by two radiologists on a separate workstation

Immediately after the 2-D examination, the same radiologist performed freehand 3-D US using a linear matrix array transducer (X6 - 1; Fig. 1). For this purpose, all patients were placed in a prone position, which typically allows for better visualization of the upper and lower renal borders [15]. First, image parameters were adjusted using pediatric presets optimized by the manufacturer. Upon activation of the 3-D X-plane mode, live sagittal and axial views of the kidney were displayed side-by-side to ensure complete renal coverage within the 3-D volume. The acquisition scan of a single dataset for each kidney took 2 s to 3 s. The multiplanar reformatted (MPR) display demonstrated the structure in three orthogonal planes along with a volume-rendered image to verify full organ inclusion. Three consecutive volumetric datasets were acquired per kidney within 1 min to 3 min. The 3-D datasets were automatically stored on the device’s internal hard drive.

Semi-automated 3-D kidney segmentation was performed on a separate workstation using prototype research software (Philips Health Technology Innovation, Paris, France), which applies implicit organ shape deformation [16]. The principle is to deform a 3-D template shape according to internal and external forces governed by fixed parameters tuned for 3-D US of renal volumes. After the initial deformation, users could make interactive corrections to adjust the segmentation to the exact capsule borders. In the second step, a region-growing algorithm is used to interactively segment the collective system, which may comprise multiple sub-volumes. Upon completion, final segmentations and corresponding volume measurements were saved. Two radiologists (7 years of experience, M.E.; 2 years of experience, A.S.) blinded to all clinical data independently delineated the kidneys on anonymized images. For each kidney, they selected the dataset that exhibited the best surface coverage and minimal motion artifacts from the three available acquisitions.

For renal capsule segmentation, a sphere was placed in the axial view to define the center of the organ, initializing the size and orientation of the 3-D US template shape, which was then deformed. Additional anchor points were placed at the superior and inferior renal poles in the sagittal view to adjust the ellipsoid capsule. The intra- and extrarenal portions of the (dilated) collective system, including calices, were segmented in a dedicated editing mode. The resulting volumes were visually checked in the three orthogonal planes. Quantitative outcomes were shown in a separate table. The following values were calculated: collective system, renal parenchyma, total kidney volume, hydronephrosis index (defined as renal parenchyma divided by total renal capsule, excluding the extrarenal proportion of the pelvis).

### Statistics

Sample size estimation was performed a priori to ensure sufficient statistical power for assessing the reproducibility of 3-D US measurements. The primary metric was the interrater reliability, expressed as the intra-class correlation coefficient (ICC). Following the method described by Zou et al. [17], the required sample size was calculated based on an expected ICC of 0.90 with a lower limit of 0.80, four raters (k = 4), and a significance level of α = 0.05 with β = 0.1 (Power = 90%). This calculation yielded a required minimum of 48 observations.

Statistical analysis was performed using IBM SPSS Statistics (version 28.0 for Windows, IBM Corp., Armonk, NY). The Kolmogorov–Smirnov test was used to study the distribution of quantification data. Continuous variable data are presented as means ± standard deviations. Data that did not follow a normal distribution are presented as median with interquartile ranges (IQR).

Bland–Altman plots were employed to visually compare renal parenchymal measurements of the two readers. The mean difference and the upper and lower limits of agreement (defined as the mean difference ± 1.96 standard deviations of the differences) were calculated.

To assess the interrater agreement, ICC values and corresponding 95% confidence intervals (CI) were obtained using an absolute agreement, two-way mixed-effects model [18]. The ICC was interpreted as slight (0–0.20), fair (0.21–0.40), moderate (0.41–0.60), substantial (0.61–0.80), and excellent (0.81–1.00). Spearman’s rank correlation test was adopted to analyze the relationship between hydronephrosis grading and the collective system volumes. A *P*-value less than 0.05 was considered to indicate statistical significance.

Dice similarity coefficients (DSC) were calculated to assess the voxel-wise spatial overlap of the 22 segmentations where both readers selected the same dataset [19]. The DSC values range from 0 (no overlap) to 1 (complete overlap).

## Results

### Patient characteristics

Forty-eight kidney volumes were evaluated in 45 children (median age, 4 years and a half; range, 1 month to 16 years; interquartile range, IQR, 7 years; 7 patients under the age of 1 year; 23 patients younger than 5 years; 35 males). The left side (*n* = 29, 60%) was more frequently affected compared to the right (*n* = 19, 40%). Detailed patient characteristics and clinical data are shown in Table 1. The most frequent hydronephrosis was grade 2 (*n* = 29), followed by grade 3 (*n* = 15). Only four children were included with grade 1 hydronephrosis.

**Table 1:** Demographic and clinical data for all included examinations

Exam	Age (y)	Sex	HN grading	max. 2-D APD (mm)	Side	Kidney disease	History of renal surgery
1	8	m	3	10	Right	Vesicorenal reflux	None
2	1	f	2	7	Left	Congenital hydronephrosis	None
3	7	m	1	8	Right	Pelviureteric junction stenosis	Pyeloplasty
4	11	f	2	13	Right	Vesicorenal reflux	None
5	11	f	2	13	Left	Vesicorenal reflux	None
6	2	f	3	15	Left	Pelviureteric junction stenosis	None
7	0.6	m	3	17	Left	Pelviureteric junction stenosis	None
8	11	m	3	22	Left	Primary obstructive megaureter	None
9	0.1	m	2	7	Right	Primary obstructive megaureter	None
10	0.1	m	3	11	Left	Primary obstructive megaureter	None
11	3	m	2	10	Left	Vesicorenal reflux	None
12	11	m	3	26	Right	Renal infundibular stenosis	None
13	10	m	2	19	Left	Pelviureteric junction stenosis	Pyeloplasty
14	0.4	m	2	7	Left	Congenital Hydronephrosis	None
15	7	m	2	10	Left	Pelviureteric junction stenosis	Pyeloplasty
16	8	m	3	15	Left	Pelviureteric junction stenosis	None
17	9	f	2	10	Right	Vesicorenal Reflux	None
18	2	m	3	14	Right	Pelviureteric junction stenosis	Pyeloplasty
19	16	m	2	21	Left	Pelviureteric junction stenosis	None
20	3	m	2	6	Right	Pelviureteric junction stenosis	Pyeloplasty
21	13	f	2	10	Left	Urogenitalis sinus	None
22	8	m	1	6	Left	Duplex kidney	None
23	1	m	3	14	Right	Pelviureteric junction stenosis	None
24	2	m	3	10	Right	Primary obstructive megaureter	None
25	4	m	1	4	Left	Duplex kidney	Upper pole resection
26	3	m	2	10	Left	Pelviureteric junction stenosis	Pyeloplasty
27	5	m	2	15	Right	Pelviureteric junction stenosis	Pyeloplasty
28	9	m	2	16	Left	Pelviureteric junction stenosis	Pyeloplasty
29	1	m	2	4	Right	Posterior urethral valves	None
30	2	f	2	7	Right	Duplex kidney	None
31	7	f	3	26	Left	Pelviureteric junction stenosis	Pyeloplasty
32	1	m	1	7	Right	Congenital hydronephrosis	None
33	12	m	2	7	Left	Pelviureteric junction stenosis	Pyeloplasty
34	2	f	2	9	Left	Pelviureteric junction stenosis	Pyeloplasty
35	4	m	2	9	Right	Congenital hydronephrosis	None
36	2	m	2	11	Left	Congenital hydronephrosis	None
37	3	m	3	15	Left	Pelviureteric junction stenosis	None
38	13	f	2	17	Right	Primary obstructive megaureter	Ureteral reimplantation
39	4	m	2	8	Right	Pelviureteric junction stenosis	Pyeloplasty
40	16	f	3	11	Left	Pelviureteric junction stenosis	Pyeloplasty
41	6	f	3	10	Left	Pelviureteric junction stenosis	Pyeloplasty
42	3	m	2	6	Right	Primary obstructive megaureter	None
43	0.3	m	2	9	Left	Congenital hydronephrosis	None
44	5	f	3	23	Left	Congenital hydronephrosis	None
45	10	m	2	21	Left	Pelviureteric junction stenosis	Pyeloplasty
46	6	m	2	20	Left	Posterior urethral valves	None
47	6	m	2	15	Right	Posterior urethral valves	None
48	3	m	2	9	Left	Primary obstructive megaureter	Ureteral reimplantation

*APD* anteroposterior diameter of renal pelvis,* D* dimensional, *f* female, *HN* hydronephrosis, *m* male, *max* maximum

### Interrater agreement

**Fig. 2:**
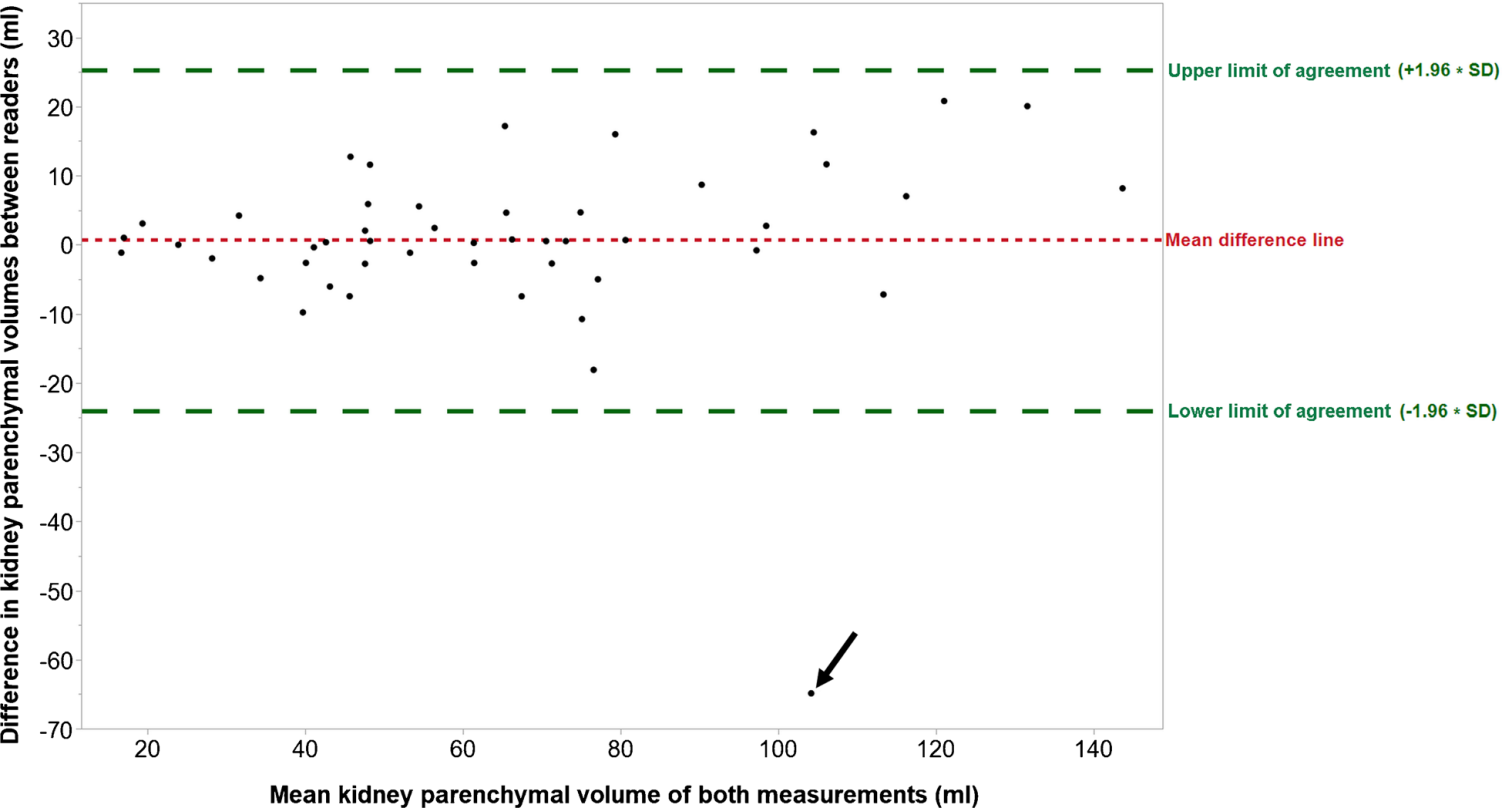
Bland-Altmann plot for all renal parenchymal values. Nearly all renal parenchymal volumes were within the limits of agreement as a tolerance range (*broken green lines*). There is a single outlier below the lower limit of agreement (*arrow*). *SD* standard deviation of the differences

The two radiologists used the same dataset for segmentation in 46% of cases (22/48). A Bland-Altmann plot illustrating the range of all renal parenchymal measurements is shown in Fig. 2. The mean difference was 0.68 ml, with upper and lower limits of agreement at 25.3 ml and − 24 ml, respectively. One outlier was identified below the lower limit of agreement: an 11-year-old child with grade 3 hydronephrosis due to renal infundibular stenosis showed a difference of 65 ml in renal parenchymal volume between the two readers. Although similar volumes were obtained for the collective system (39.6 ml and 40.4. ml), a marked discrepancy was observed in the segmentation of the renal capsule (111 ml vs 177 ml). In this case, different datasets were used for kidney segmentation. The retrospective manual comparison revealed that the dataset used by reader 1 did not fully include the upper renal pole. Given the limited number of cases in each subgroup, the mean absolute difference in renal parenchymal volume between the two readers was 5 ml for hydronephrosis grade 1, 6.8 ml for grade 2, and 4.7 ml for grade 3 (after exclusion of the identified outlier).

Interrater agreement was good to excellent across all metrics:

- Total renal volume (renal capsule): ICC, 0.94; 95% CI [0.9; 0.97]; *P* < 0.001
- Collective system: ICC, 0.87; 95% CI [0.78; 0.93]; *P* < 0.001
- Hydronephrosis index: ICC, 0.83; 95% CI [0.71; 0.9]; *P* < 0.001
- Renal parenchyma ICC, 0.92; 95% CI [0.86; 0.96]; *P* < 0.001

The median renal parenchymal volumes were 62.6 ml and 62 ml for the two readers, ranging from 16.1 ml to 147.7 ml (reader 1; IQR, 37.9 ml) and from 16.5 ml to 139.5 ml (reader 2; IQR 41.3 ml). The median hydronephrosis index was 0.91 for both readers, ranging from 0.63 to 0.99 (reader 1; IQR, 0.10) and 0.52 to 0.98 (reader 2; IQR 0.12).

In the 22 delineations using the same dataset, DSCs were as follows:

- Total renal volume (capsule): median DSC, 0.90; range, 0.76–0.95; IQR, 0.06
- Collective system: median DSC, 0.77; range 0.54–0.89; IQR, 0.76
- Renal parenchyma: median DSC, 0.88; range 0.73–0.94; IQR, 0.10)

**Fig. 3:**
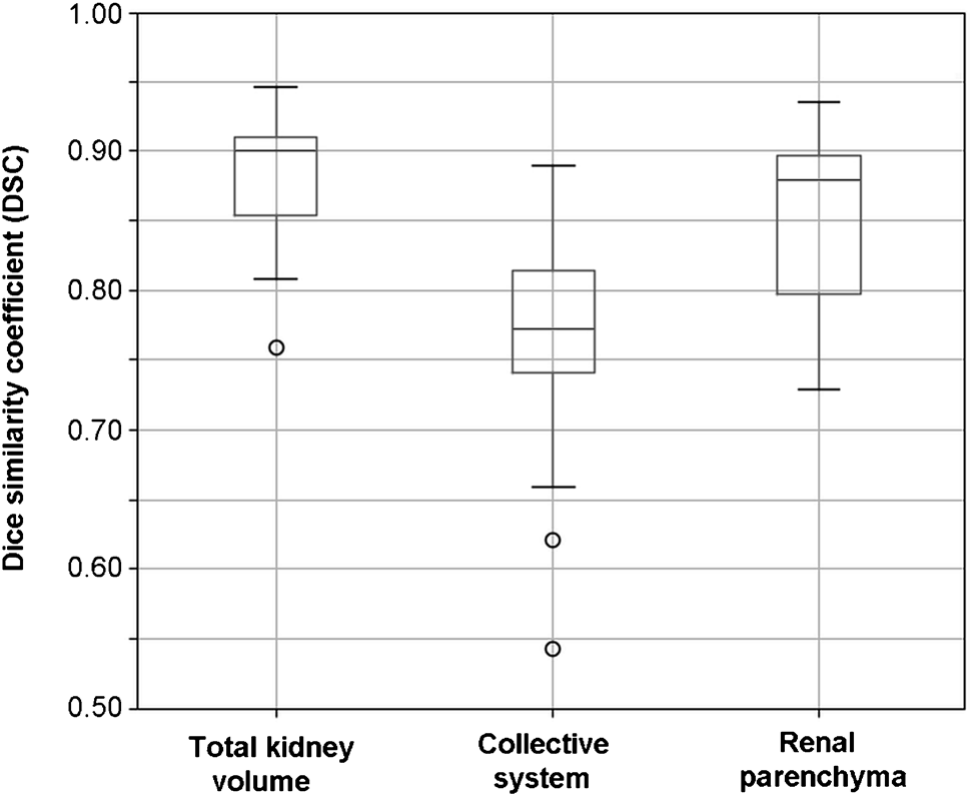
Boxplots of Dice similarity coefficients for total renal volume, collective system, and parenchyma. The highest median values (*bold lines*) of Dice similarity coefficient (DSC) are found for total renal volume (capsule: median, 0.9) and for the parenchyma (median: 0.88). The collective systems show lower DSCs (median: 0.77) with a relatively wide range (from 0.54 to 0.89). The Dice values for renal parenchyma segmentation are lower than those for capsule segmentation but remain high. Since the delineation of the renal capsule is semi-automatic, its high spatial agreement seems to compensate for the discrepancies in the collective system segmentation. This can also be explained by the fact that the volume of the latter is generally much smaller than that of the former

The corresponding boxplots for all DSCs are presented in Fig. 3. A remarkably low DSC was observed for the collective system (DSC, 0.54) in an 8-year-old male with a left-sided duplex kidney. Similar absolute values were obtained for the volumes of the slightly dilated renal pelvis (grade 1; 1.4 ml vs 1.5 ml), although the spatial overlap between segmentations was not optimal. This also resulted in a comparable renal parenchymal volume with an interreader difference of 4.7 ml (77 ml vs 73 ml).

### Hydronephrosis grading

The median maximum anteroposterior diameter of the renal pelvis was 10 mm (range, 4–26 mm; IQR, 7 mm). There was a positive correlation between the anteroposterior diameter and hydronephrosis grading (*P* < 0.001), between anteroposterior diameter and the hydronephrosis index (*P* < 0.001), and between anteroposterior diameter and the volume of the collective system (*P* < 0.001).

**Fig. 4:**
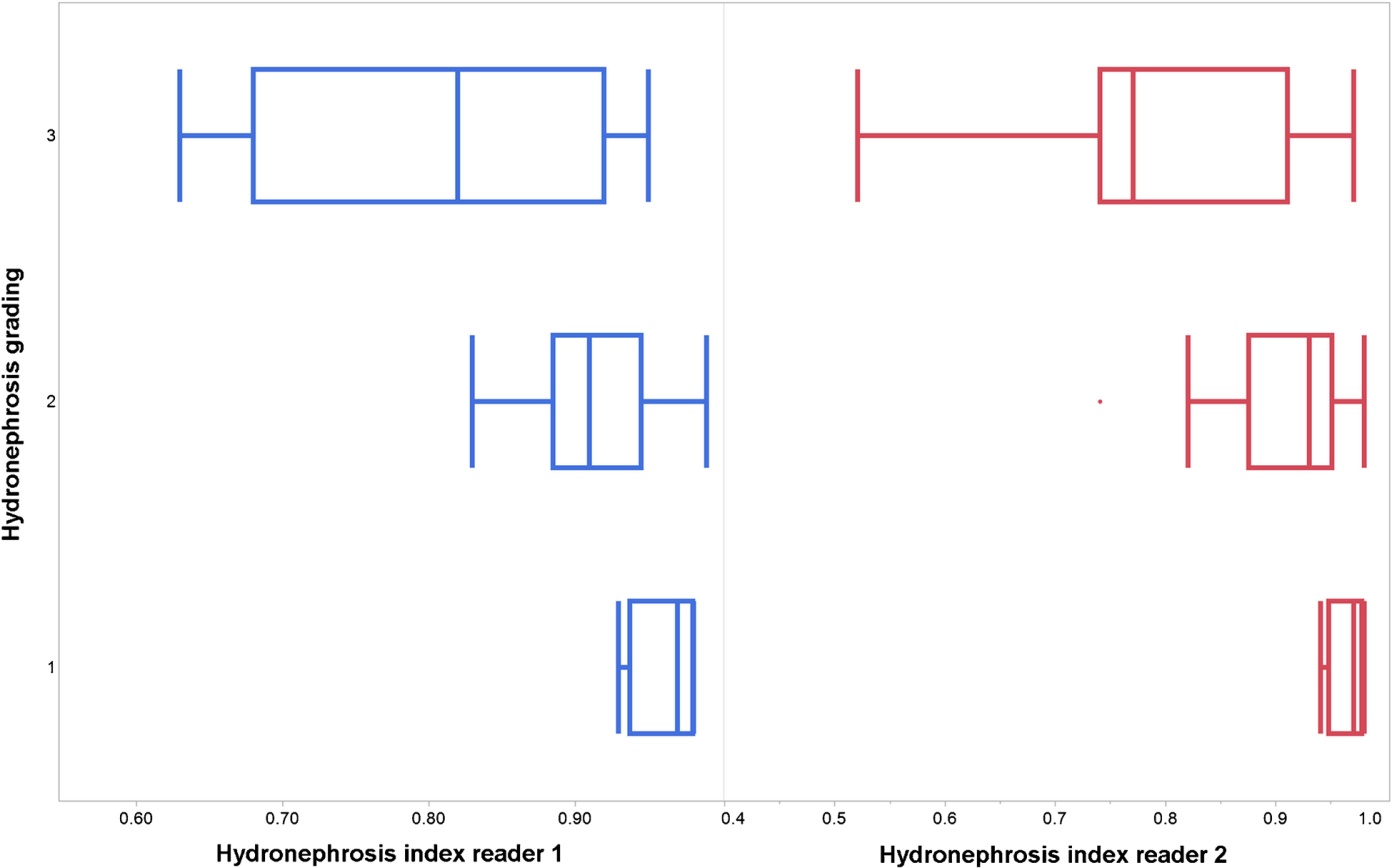
Correlation between hydronephrosis indices and clinical grading. Lower values of the hydronephrosis index (renal parenchyma divided by total capsule) are associated with a higher degree of hydronephrosis (*P* < 0.001)

The hydronephrosis index had a median of 0.91 for both readers (IQR, 0.1 for both) with ranges of 0.6 to 1 (reader 1) and 0.5 to 1 (reader 2). There was a positive correlation between hydronephrosis grading and both the hydronephrosis index (*P* < 0.001; Fig. 4) and the volume of the collective system (*P* < 0.001).

## Discussion

This study provides initial data concerning the feasibility of freehand 3-D renal volume assessment to evaluate the severity of hydronephrosis in children. The presented workflow allowed for reproducible and accurate volumetric measurements, offering helpful information according to established grading methods. Measurements can be obtained rapidly and easily without exposure to radiation, contrast agents, or the need for anesthesia. Therefore, this method is suitable for all pediatric patients, supporting its feasibility for routine renal parenchymal assessment in clinical practice. As part of the presented workflow, the prototype software for semiautomatic segmentation (Fig. 1) allowed a reliable subtraction of the dilated collecting system and enables reproducible calculation of renal parenchymal volume.

A recent review recognized 3-D US as the “least invasive and most cost-effective way” to monitor children’s renal size, emphasizing its importance for future research [2]. Early clinical studies have demonstrated superior accuracy in depicting renal anatomy and reliably assessing parenchyma volume in children with malformations and post-operative changes compared to planimetric analysis [7, 9]. In our study, a substantial portion of the cohort presented with congenital renal anomalies (Table 1) and/or complex urinary tract anatomy, such as urogenital sinus or duplex kidney. Additionally, prior renal surgery had altered the anatomy in 18 patients (38%). Despite these complexities, our results support using matrix array 3-D US as a robust imaging technique, particularly in cases with anatomical variations.

As the anteroposterior diameter is commonly used as a proxy for hydronephrosis severity, our data suggest that parenchymal volume could serve as a complementary indicator, particularly in borderline cases where anteroposterior diameter alone may not be conclusive. In line with findings from Onen et al., who correlated hydronephrosis grades with surgical indications, we propose that integrating volumetric analysis could improve the accuracy of assessing intervention needs and timing [1]. While our study was not designed to predict surgical outcomes, it highlights the potential of volumetric assessment in refining prognostic evaluation. Future research should explore its role in clinical decision-making, particularly by correlating 3-D US-derived parenchymal volumes with split renal function, as measured by diuretic scintigraphy or MR urography.

A recent study recommended 3-D US over MRI for renal volume assessment in the follow-up of children with autosomal dominant polycystic kidney disease [8]. In contrast to our study, it excluded hydronephrotic kidneys and included only older patients (> 8 years). In contrast, our cohort included a large proportion of young patients (51% under 5 years, including seven infants), who are especially prone to movement during 3-D acquisition. This highlights the advantage of fast volume capture using a matrix array transducer in this age group. In the comparative study of Fritz et al., infants younger than 6 months were included; however, six were excluded from the analysis because of increased motion, resulting in unusable images [9]. Riccabona et al. similarly reported that 3-D US was impossible in some uncooperative infants [3]. In our prospective study, no child was excluded, and the software evaluated at least one of the three acquired datasets for all patients.

Riccabona et al. assessed hydronephrotic kidneys using an electromagnetic positioning device and a mechanically driven probe but did not utilize an electronic matrix array transducer. [3]. In a similar study by Fritz et al., only eight patients with hydronephrosis were analyzed [9]. Both studies [3, 9] used semiautomated volume calculation with threshold-based segmentation. Our study applied a comparable model to minimize time requirements. Our evaluation demonstrated that accurate identification of renal margins is pivotal for reliable parenchymal volume assessment. Since the renal capsule volume is much larger than the renal pelvis, minor inaccuracies in pelvic measurements can be compensated. This may explain why one child with a significant discrepancy in renal capsule volume between the readers was an outlier in the parenchymal volume comparison (Fig. 2). The choice of which dataset to segment could also play a role here: in the case mentioned above, two different datasets were segmented. We consider it advantageous that in routine practice with uncooperative children (who are unable to hold their breath), having multiple 3-D acquisitions provides greater certainty for accurate segmentation-provided both poles have been fully captured in all datasets. The time required for multiple acquisitions is minimal when using a matrix transducer.

Initial studies evaluating matrix array transducers for renal volume calculation in adults compared normal renal volumes with CT [12] and investigated correlations with function parameters, but they did not assess reproducibility [11]. Both studies valued 3-D US with a matrix array transducer as a reliable tool for organ volume measurement with reduced errors and recommended evaluating the reproducibility in different clinical cohorts [12]. The only report on a matrix array probe in hydronephrosis included eight kidneys in a feasibility study of a new segmentation model [14]. The renal volumes were analyzed concerning segmentation errors and relative differences, but the interrater variability was not assessed.

In earlier research, the hydronephrosis index has been proposed as a dimensionless parameter for quantifying hydronephrosis in children using 2-D US [20]. The benefits of standardized measurements have been previously emphasized [21]. Rud et al. reported excellent interobserver agreement and a correlation between hydronephrosis index and the sonographic degree of renal pelvis dilation in adults with stone-related renal colic [22]. Another study showed close correlation between the hydronephrosis index and sonographically evaluated hydronephrosis grade in patients with pelvi-ureteric junction obstruction [23]. These conclusions align with our findings, which demonstrate a good interrater agreement (ICC 0.83) and a strong correlation between the hydronephrosis index and both the hydronephrosis grading (*P* < 0.001; Fig. 4) and the 2-D measurement of the anteroposterior diameter (*P* < 0.001). Han et al. found that pre-operative hydronephrosis index in patients for pyeloplasty can work as a possible prognostic marker for adverse renal function outcomes [24]. Although we did not evaluate the clinical value of 3-D US parameters, our findings suggest that semiautomated calculation of the hydronephrosis index from 3-D datasets as an indicator for pediatric renal function offers a simplified and objective measurement approach, meriting further investigation. It is worth noting that 2-D sonographic calculation of the index can be time-consuming [1].

Our study has the following limitations: Unlike previous studies, we did not compare our results to CT, MR, or 2-D US. However, the general feasibility and advantages of 3-D US over conventional methods have already been published. Additionally, we used a transducer with a frequency range of 6–1 MHz in all children, which resulted in low image resolution in infants and neonates. Nonetheless, complete coverage of the renal capsule was thus easily possible without the need to interpolate upper and/or lower pole contours. Image contrast was estimated sufficient for identifying the collective system in all patients. No cases of grade 4 hydronephrosis were included, which may limit the generalizability of the results; however, we assume that volumetric analysis would not be significantly affected, and the general trends observed in our study would remain valid even if the analysis were restricted to high-grade hydronephrosis.

## Conclusion

Novel semiautomatic 3-D US volumetric analysis has a high degree of interrater agreement and enables reliable assessment of renal parenchymal volume in hydronephrosis. Volumes of the collective system and the hydronephrosis index correlate with the extent of hydronephrosis.

## Data Availability

The data that support the findings of this study are available from Philips Healthcare. Restrictions apply to the availability of these data, which were used under license for this study.
